# Artepillin C Reduces Allergic Airway Inflammation by Induction of Monocytic Myeloid-Derived Suppressor Cells

**DOI:** 10.3390/pharmaceutics13111763

**Published:** 2021-10-22

**Authors:** Núbia Sabrina Martins, Thais Fernanda de Campos Fraga-Silva, Giseli Furlan Correa, Mèdéton Mahoussi Michaël Boko, Leandra Naira Zambelli Ramalho, Débora Munhoz Rodrigues, Juliana Issa Hori, Diego Luis Costa, Jairo Kenupp Bastos, Vânia Luiza Deperon Bonato

**Affiliations:** 1 Basic and Applied Immunology Program, Ribeirao Preto Medical School, University of Sao Paulo, Ribeirao Preto 14049-900, Sao Paulo, Brazil; nsmartins@usp.br (N.S.M.); bokmich@usp.br (M.M.M.B.); dlcosta@usp.br (D.L.C.); 2 Department of Biochemistry and Immunology, Ribeirao Preto Medical School, University of Sao Paulo, Ribeirao Preto 14049-900, Sao Paulo, Brazil; thaisfragasilva@usp.br (T.F.d.C.F.-S.); giselifurlan@usp.br (G.F.C.); 3 Department of Pathology and Legal Medicine, Ribeirao Preto Medical School, University of Sao Paulo, Ribeirao Preto 14049-900, Sao Paulo, Brazil; lramalho@fmrp.usp.br; 4 Department of Pharmaceutical Sciences, School of Pharmaceutical Sciences, University of Sao Paulo, Ribeirao Preto 14049-900, Sao Paulo, Brazil; debora_munhoz@yahoo.com.br (D.M.R.); jkbastos@fcfrp.usp.br (J.K.B.); 5 Apis Flora Industrial and Comercial Ltda, Ribeirao Preto 14020-670, Sao Paulo, Brazil; julianabio2000@gmail.com

**Keywords:** propolis, artepillin C, allergic asthma, M-MDSC, therapies

## Abstract

Propolis is a natural product produced by bees that is primarily used in complementary and alternative medicine and has anti-inflammatory, antibacterial, antiviral, and antitumoral biological properties. Some studies have reported the beneficial effects of propolis in models of allergic asthma. In a previous study, our group showed that green propolis treatment reduced airway inflammation and mucus secretion in an ovalbumin (OVA)-induced asthma model and resulted in increased regulatory T cells (Treg) and polymorphonuclear myeloid-derived suppressor cells (PMN-MDSC) frequencies in the lungs, two leukocyte populations that have immunosuppressive functions. In this study, we evaluated the anti-inflammatory effects of artepillin C (ArtC), the major compound of green propolis, in the context of allergic airway inflammation. Our results show that ArtC induces in vitro differentiation of Treg cells and monocytic MDSC (M-MDSC). Furthermore, in an OVA-induced asthma model, ArtC treatment reduced pulmonary inflammation, eosinophil influx to the airways, mucus and IL-5 secretion along with increased frequency of M-MDSC, but not Treg cells, in the lungs. Using an adoptive transfer model, we confirmed that the effect of ArtC in the reduction in airway inflammation was dependent on M-MDSC. Altogether, our data show that ArtC exhibits an anti-inflammatory effect and might be an adjuvant therapy for allergic asthma.

## 1. Introduction

Propolis is a resin made by working *Apis mellifera* Linnaeus, 1758 bees from exudates and new buds of different plant species, which is traditionally used in alternative and complementary medicine. The Brazilian green propolis has the shrub *Baccharis dracunculifolia* DC as its primary botanical source and is rich in prenylated derivative compounds. It has gained notoriety for displaying anti-inflammatory, immunomodulatory, antitumoral, antibacterial, and antiviral proprieties [1,2,3,4]. Experimental evidence of anti-inflammatory actions of propolis was demonstrated in several murine models of inflammatory diseases. Those actions include the reduction in neutrophils and the levels of pro-inflammatory cytokines IL-6 and TNF-α in the bronchoalveolar lavage fluid (BALF) during LPS-induced pulmonary inflammation [5]; reduction in neutrophil infiltration and serum levels of IL-6, IL-1β and TNF-α in experimental pancreatitis [6], and decreased production of reactive oxygen species (ROS) in ethanol-induced gastric ulcers [7]. In the type 2 inflammation induced by inhaled allergen, the treatment with propolis during the sensitization phase resulted in reduction in allergen-specific IgE, airway inflammation, and hyper-reactivity [8] and in a decreased number of inflammatory cells in the BALF [9]. Our research group characterized the anti-inflammatory action of green propolis in sensitized and challenged mice and showed a reduction in pulmonary inflammation and mucus secretion [10], suggesting a protective effect for propolis treatment in asthma.

Asthma is a chronic inflammatory lung disease that affects 339 million people worldwide [11]. Bronchial hyper-reactivity, mucus overproduction and airway remodeling induce symptoms such as shortness of breath, cough, and wheezing [12,13]. Although there are different disease phenotypes, the hallmark of allergic asthma is a type 2 immune response, characterized by IL-4, IL-5, and IL-13 secretion by T helper 2 (Th2) lymphocytes, IgE secretion, recruitment and activation of eosinophils, mast cells, dendritic cells and epithelial cells [12]. Currently, therapy with corticoids configures the most efficient treatment to control the disease symptoms but it does not cure asthma. Moreover, the recurrent use of corticoids favors the development of infections and impairs childhood development [12,14]. Therefore, there is an urgent need for new therapies for allergic asthma, both to control disease symptoms as well as to improve the life quality of asthmatic patients. 

Although propolis treatment represents a promising strategy for immunotherapy, it is also challenging considering the isolation of the compounds that display the anti-inflammatory effects. Artepillin C (ArtC) (3,5-Diprenyl-4-hydroxicinnamic) is a prenylated phenolic compound derived from cinnamic acid and is one of the main components in green propolis [15]. Some in vitro studies demonstrated that ArtC inhibits ROS production, cytokine secretion and blocks NF-κB expression in IFN-γ-stimulated RAW264.7 macrophage cell lineage [16]. Additionally, ArtC exhibits an anti-inflammatory role in an experimental model of peritonitis, in which it induces reduction in leukocyte and PGE_2_ levels in peritoneal exudate [17].

In this study, we aimed to evaluate the anti-inflammatory activity of ArtC in allergic airway inflammation. We hypothesized that ArtC induces regulatory CD4^+^ T (Treg) cells and myeloid-derived suppressor cells (MDSC), which negatively regulates allergic pulmonary inflammation. Treg cells are involved in the regulation of immune response by suppressing the function of effector T cells (Th1, Th2, Th17, and CD8^+^ T cells) [18]. MDSC comprises a heterogeneous cell population derived from bone marrow that expands in situations of physiological stress, such as chronic inflammation and tumors, and also display immunosuppressive actions [19,20,21]. There are two subsets of MDSC: the granulocytic (PMN-MDSC) and the monocytic (M-MDSC), which are classified according to their origin, either from granulocytic or monocytic myeloid cells lineages [22,23].

We showed that ArtC treatment reduced eosinophilic lung inflammation in an ovalbumin (OVA)-induced allergic asthma model and induced M-MDSC differentiation in vivo and in vitro. Finally, we performed ArtC treated-M-MDSC transfer to animals sensitized and challenged with the allergen and confirmed that the decrease in eosinophilic lung inflammation was dependent on ArtC.

## 2. Materials and Methods

### 2.1. Artepillin C (ArtC) Isolation

ArtC was isolated from the crude extract of green propolis provided by Apis Flora Company (Ribeirao Preto, SP, Brazil). First, the green propolis was frozen, pulverized in a blender, and extracted with hydroalcoholic solution (9:1) to furnish the crude hydroalcoholic extract. It was fractioned using a silica gel open chromatographic column with a gradient elution consisting of hexane–ethyl acetate 95:05 to 90:10 *v/v* furnishing approximately 100 fractions. Each fraction was analyzed by TLC (thin-layer chromatography), concentrated under vacuum, and combined according to its chromatographic similarities. The fractions were analyzed by HPLC-DAD (high-performance liquid chromatography with a diode-array detector) using authentic ArtC standard. Then, the fractions rich in ArtC were purified by preparative HPLC-UV at 275 nm furnishing 68 mg of pure ArtC with relative purity of 95%. The elution program consisted of 7 min gradient with 80% B (methanol) and 20% A (99% water, 1% formic acid), 12 min with 85%B, 20 min with 90% B, 22 min with 95% B, and a flow rate of 8.0 mL/min, and detection at 275 nm, using a reverse-phase column (Shimadzu Shim-pack prep.-ODS 15 µm, 20 × 250 mm) [24]. ArtC chemical structure was confirmed by spectroscopic (1H NMR), and spectrometric (High-resolution ESI-MS) analyses. ArtC was solubilized in DMSO and maintained at −80 °C until the moment of use. For in vivo experiments, ArtC-DMSO was diluted in phosphate buffered sSaline (PBS) with a final concentration of 0.05% of DMSO. For in vitro experiments, ArtC-DMSO was diluted in RPMI 1640 (Sigma-Aldrich, St. Louis, MO, USA) with a final concentration ≤1.8% DMSO.

### 2.2. Animals

Female C57BL/6 mice (6–8 weeks old) were obtained from the breeding facility of Ribeirao Preto Medical School, University of São Paulo (Ribeirao Preto, SP, Brazil). All animals were maintained in sterile environmental conditions in a ventilated rack (Alesco, Monte Mor, SP, Brazil) and received sterile food and water. Body weight was measured weekly. All experiments were performed according to the local Ethics Committee on Animal Experimentation (Protocol Number 216/2019).

### 2.3. OVA-Induced Asthma Model

Mice were sensitized three times with 10 μg of ovalbumin (OVA) Grade-VI emulsified in 2 mg of aluminum hydroxide (alum) (all from Sigma-Aldrich, St. Louis, MO, USA) by intraperitoneal route with seven-day intervals. Mice were challenged three consecutive days with 30 μg of OVA Grade V (Sigma-Aldrich, St. Louis, MO, USA) by intranasal route seven days after the third sensitization. For challenge, mice were anesthetized with ketamine (100 mg/kg, Sespo Industry and Commerce, Paulínia, SP, Brazil) and xylazine (10 mg/kg, Vetecia Laboratory of Veterinary Products, Jacareí, SP, Brazil).

### 2.4. Treatment

Mice were treated with seven doses of vehicle (PBS, 0.05% DMSO) or ArtC (80 μg, 0.05% DMSO) by intranasal route 24 h after the third allergen challenge in alternate days. After the end of treatment, the mice were challenged with the allergen three more times. For challenge, mice were anesthetized with ketamine (100 mg/kg, Sespo Industry and Commerce, Paulínia, SP, Brazil) and xylazine (10 mg/kg, Vetecia Laboratory of Veterinary Products, Jacareí, SP, Brazil).

### 2.5. Bronchoalveolar Lavage

To assess bronchoalveolar lavage fluid (BALF), animals were anesthetized with ketamine (100 mg/kg, Sespo Industry and Commerce, Paulínia, SP, Brazil) and xylazine (10 mg/kg, Vetecia Laboratory of Veterinary Products, Jacareí, SP, Brazil). Then, the trachea was exposed and cannulated with Angiocath with subsequent injection of 4 mL of PBS. Next, samples were centrifuged at 450× *g* for 5 min. The supernatant was stored at −20 °C for cytokine measurement. Cells were resuspended in 500 μL of RPMI 1640 (Sigma-Aldrich, St. Louis, MO, USA), centrifuged at cytocentrifuge (Thermo Fisher Scientific, Waltham, MA, EUA) at 18× *g* for 3 min, and stained with rapid panoptic (Labor Clin, Sao José do Rio Preto, SP, Brazil) for differential cell count.

### 2.6. Lung Homogenate

After collection of BALF, animals were perfused with 5 mL of PBS. Left lung lobules were mechanically macerated in microtubes containing protease inhibitor (Sigma-Aldrich, St. Louis, MO, USA) and centrifuged at 3220× *g* for 15 min. The supernatant was collected and maintained at −20 °C for cytokine measurement.

### 2.7. Histopathology

The histopathological analysis was performed as previously described [25]. Briefly, right lung lobules were stained with hematoxylin and eosin (H&E) to evaluate the inflammatory infiltrate and periodic acid-Schiff (PAS) to evaluate mucus production. Histological images were performed using the objective lens of 20× (magnification 200×). Pulmonary inflammation score was determined following the criteria: 0–without inflammation; 1—mild inflammation, and 2—moderate to severe inflammation. Mucus score was determined following the criteria: 0—without mucus production, and 1—mild mucus production.

### 2.8. MDSC Differentiation

Bone marrow-derived MDSC were generated according to Solito and colleagues [26] from female C57BL/6 mice. Briefly, cells were stimulated with recombinant IL-6 (40 ng/mL, BD Pharmingen, San Diego, CA, USA) and GM-CSF (40 ng/mL, BD Pharmingen, San Diego, CA, USA) in the presence or absence of ArtC (0.01, 0.1, 5, 10, 20, 50, or 100 μM). Cells were cultured for 96 h, at 37 °C and 5% CO_2_.

### 2.9. Treg Cell Differentiation

Naive T cells (CD4^+^CD62L^+^) from spleen and lymph nodes of female C57BL/6 mice, purified using magnetic beads (Mylteni Biotec, Bergisch Gladbach, NRW, Germany), were distributed in plates previously coated overnight with monoclonal antibody anti-CD3 (5 μg/mL, BD Pharmingen, San Diego, CA, USA) in the presence of anti-CD28 (1 μg/mL), rTGF-β (3 ng/mL, BD Pharmingen, San Diego, CA, USA) and rIL-2 (10 ng/mL, BD Pharmingen, San Diego, CA, USA), treated or not with ArtC (0.01, 0.1, 5, 10, 20, 50 or 100 μM). For Treg cell differentiation, cells were cultured for 96 h, at 37 °C and 5% CO_2_.

### 2.10. Flow Cytometry

Lung cells were isolated by proper right lung lobules digestion using collagenase (2.2 mg/mL) (Sigma-Aldrich, St. Louis, MO, USA) and DNAse (0.055 mg/mL) (Roche, Basel, Switzerland). Characterization of MDSC and Treg cells was performed by flow cytometry according to antibodies fabricant instructions (BD Pharmingen, San Diego, CA, USA). Samples were stained with FVS780 viability stain, CD45, CD11b, Ly6C, and Ly6G to characterize MDSC and CD45, CD4, and Foxp3 to characterize Treg cells (Table 1). Samples were fixed using PBS containing 1% paraformaldehyde (Labsynth, Diadema, SP, Brazil). The samples were acquired in FACS Melody (BD Biosciences, San Jose, CA). On average, two hundred and fifty thousand events per sample were collected within the gate of viable cells (FVS780^−^). Analyses were performed in FlowJo software (Becton Dickinson and Company, Franklin Lakes, NJ, USA).

### 2.11. M-MDSC Adoptive Transfer

For adoptive transfer, MDSC were generated in the presence or absence of ArtC (10 μM). Subsequently, M-MDSC (Ly6G^−^Ly6C^+^) were purified using FACS Melody sorting (BD Biosciences, San Jose, CA, USA), and 1 × 10^5^ cells were transferred by intrapharyngeal route to OVA sensitized-mice during the second allergen challenge. Twenty-four hours after the third challenge, animals were euthanized to evaluate lung inflammation.

### 2.12. Cytokines

IL-4, IL-5, IL-10, and IL-13 levels were determined in BALF and lung homogenates using ELISA kits following the manufacturer’s instructions (R&D Systems, Minneapolis, MN, USA). The limit of detection was 31.2 pg/mL.

### 2.13. Statistical Analysis

Data were analyzed using GraphPad Prism Version 8.1 (GraphPad Software, Inc., San Diego, CA, USA). Two-group comparisons were analyzed by non-paired t-test, and three or more groups’ comparisons were calculated by ANOVA one-way followed by Tukey’s test. Correlation analyses were performed following Pearson’s correlation coefficient. The histological score was calculated by the Chi-square test. Data were shown as the mean ± standard deviation (SD), and the results were considered significant with a *p*-value less than 0.05.

## 3. Results

### 3.1. ArtC Isolation

**Figure 1:**
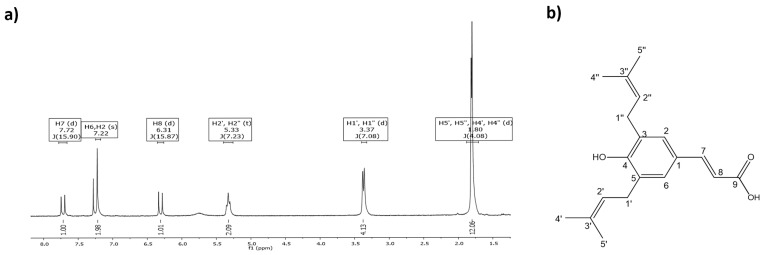
ArtC isolation. (**a**) ^1^H NMR spectrum of ArtC (CD3OD, 300 MHz); (**b**) chemical structure of ArtC.

The chromatographic processes used to obtain pure ArtC were previously reported by Rodrigues and colleagues [24] and yielded approximately 68 mg of purified compound, which was used for running the biological assays. ArtC chemical structure was confirmed by ^1^H NMR (CDCl_3_, 300 MHz) δH: 7.72 (d, 1H, J 15.9 Hz, H7), 7.22 (s, 2H, H6 and H2), 6.31 (d, 1H, J 15.9 Hz, H8), 5.33 (t, 2H, J 7.2 Hz H2′, and H2″), 3.37 (d, 4H, J 15.9 Hz, H1′, and H1″), and 1.80 (d, 12H, H5′, H5″, H4′, and H4″). For C_19_H_24_O_3_ [M + H]^+^: 301.1759 (Figure 1a); found C_19_H_24_O_3_ [M + H]^+^: 301.1790 (Figure 1b).

### 3.2. ArtC Attenuates Allergic Airway Inflammation

**Figure 2:**
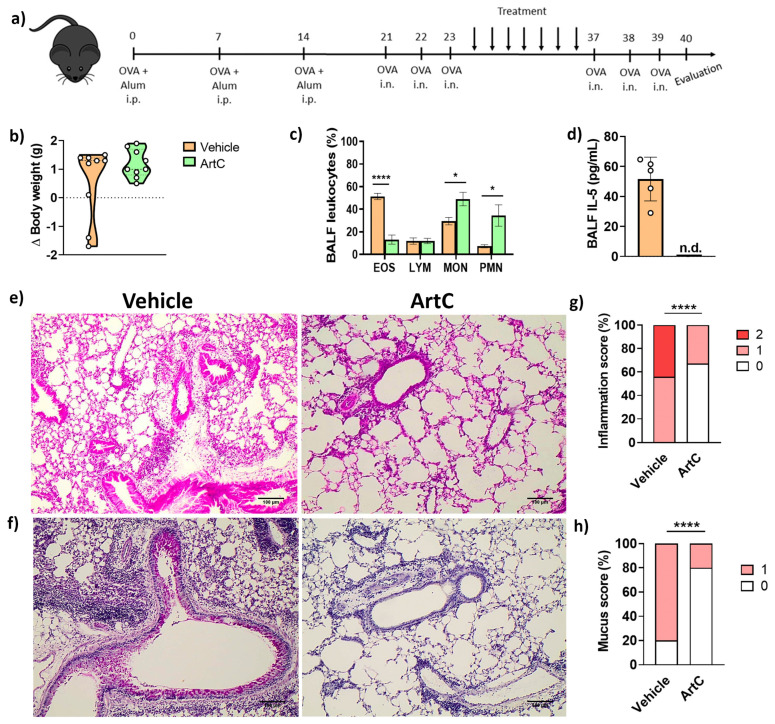
ArtC reduces allergic airway inflammation. (**a**) Experimental design; (**b**) body weight variation of mice exposed to the allergen before and after treatment with ArtC; (**c**) frequency of eosinophils (EOS), lymphocytes (LYM), mononuclear cells (MON) and polymorphonuclear cells (PMN) in the BALF; (**d**) IL-5 in the BALF (n.d.: not detected); (**e**) representative images of lung inflammation (magnification 200×, scale bar 100 µM) and (**f**) mucus production (magnification 200×, scale bar 100 µM); (**g**) lung inflammation score (0—without inflammation; 1—mild; 2—moderate to severe inflammation); (**h**) mucus score (0—without mucus secretion; 1—mild). Data are representative of two independent experiments (n = 4–5/group/experiment), except for (**d**) (one representative experiment), and expressed by mean ± SD. * *p* < 0.05 and **** *p* < 0.0001.

We and others have previously demonstrated the anti-inflammatory effect of propolis in experimental allergic asthma [8,9,10,27]. However, a major challenge for the development of immunotherapies employing natural products, such as propolis, is to identify the individual active compounds that display anti-inflammatory activities. In order to do that, OVA-sensitized and challenged mice were treated with ArtC (treated group) or with vehicle (PBS, non-treated group), as depicted in Figure 2a. Following treatment, mice were challenged with the allergen and lung inflammation was evaluated (Figure 2a). No differences in body weight were detected prior to and after treatment between treated and non-treated groups (Figure 2b). 

There was a significant reduction in the frequency of eosinophils and IL-5 levels along with an increase in mono and polymorphonuclear cells in the BALF of ArtC-treated animals compared with that of non-treated allergen-exposed mice **(**Figure 2c,d). No difference was found in IL-4 concentrations in lung homogenates and no IL-4 was detected in the BALF comparing groups treated or not treated with ArtC, previously exposed to OVA (vehicle group and ArtC group). IL-13 concentrations were also similar in the BALF and in the lung homogenates between both groups (data not shown). The histopathological analysis clearly showed a significant decrease in the perivascular and peribronchial cellular infiltrates (Figure 2e) and mucus secretion (Figure 2f) in lungs of ArtC-treated mice compared to those of animals in the vehicle-treated group. Inflammation and mucus scores were evaluated and a reduction in both parameters was observed following ArtC treatment, whereas two represented the highest level of inflammation and one the highest level of mucus production, respectively (Figure 2g,h).

Collectively, these results show that the in vivo treatment with ArtC reduced allergic airway inflammation in a model of OVA-induced asthma. 

### 3.3. ArtC Induces Treg Cells Differentiation In Vitro, but Not In Vivo

**Figure 3:**
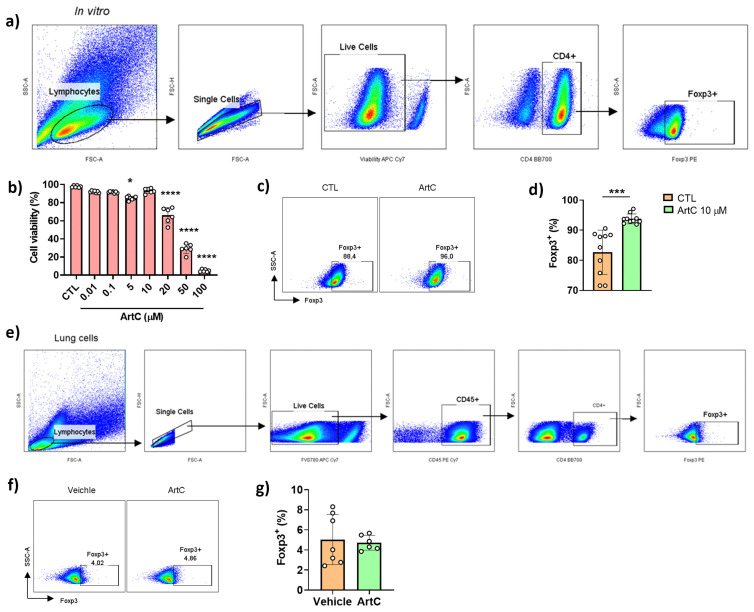
ArtC induces Treg cells differentiation in vitro. (**a**) Gate strategy for Treg cell (CD4^+^Foxp3^+^) characterization in vitro by flow cytometry; (**b**) frequency of live cells (FVS780^−^) in culture of CD4^+^ T cells stimulated with ArtC versus control (CTL); (**c**) representative dot plot of Treg cells generated in vitro in the presence or absence of ArtC; (**d**) percentage of Treg cells generated in vitro. Data are representative of three independent experiments (n = 3–4/group/experiment) and expressed by mean ± SD; (**e**) gating strategy for Treg cell characterization in vivo; (**f**) representative dot plot of Treg cell in the lungs of mice exposed to the allergen and treated or not (vehicle) with ArtC; (**g**) frequency of Treg cells in the lungs. Data are representative of two independent experiments (n = 4–5/group/experiment) and expressed by mean ± SD. * *p* < 0.05, *** *p* < 0.001 and **** *p* < 0.0001.

Because we previously reported that propolis increases the differentiation of regulatory (Treg) T cells [10], we first investigated whether purified ArtC would induce Treg cells in vitro. Treg cells were differentiated from CD4^+^CD62L^+^ naive T lymphocytes purified from spleen and lymph nodes and further cultured in the presence of recombinant IL-2 and TGF-β. Figure 3a depicts the gate strategy used to evaluate Treg cells expressing the transcription factor Foxp3. ArtC was not toxic at concentrations up to 10 µM (Figure 3b). The addition of ArtC in the latter non-toxic concentration (10 µM) increased the differentiation of Treg cells compared with those cells cultured with IL-2 and TGF-β only (CTL) (Figure 3c,d). We next evaluated the frequency of Treg cells in the lungs of ArtC-treated mice according to the gate strategy represented in Figure 3e. ArtC treatment neither affected the frequency of Foxp3^+^ Treg cells (Figure 3f) nor the production of IL-10 (data not shown) in the lungs of treated mice, indicating that although ArtC induced Foxp3^+^ Treg cells differentiation in vitro, this outcome was not observed in lungs of mice exposed to the allergen in vivo. 

### 3.4. ArtC Augments M-MDSC Frequency In Vitro and In Vivo

**Figure 4:**
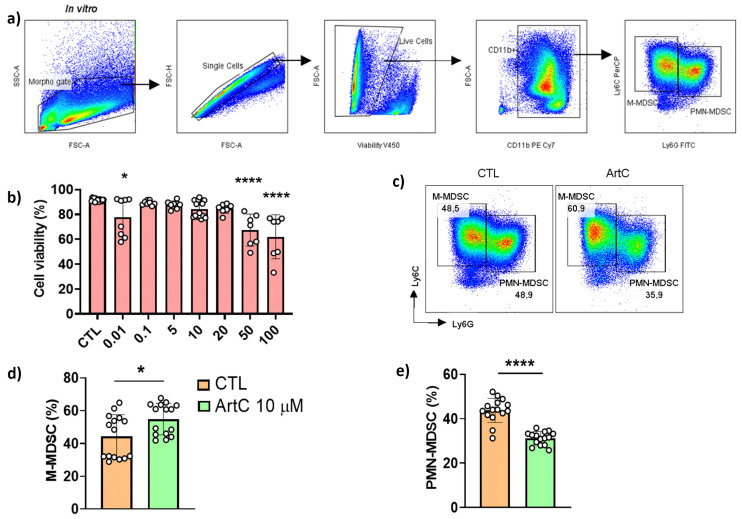
ArtC induces M-MDSC differentiation in vitro. (**a**) Gate strategy for M- (Ly6G^−^Ly6C^+^) and PMN-MDSC (Ly6G^+^Ly6C^int^) characterization in vitro; (**b**) frequency of live cells (FVS780−) in culture of bone marrow cells stimulated with rIL-6, GM-CSF and ArtC versus control (CTL); (**c**) representative dot plot of M- and PMN-MDSC generated in vitro in the presence of ArtC (10 µM); (**d**) percentage of M-MDSC, and (**e**) PMN-MDSC. Data are representative of four independent experiments (n = 3–4/group/experiment) and expressed by mean ± SD. * *p* < 0.05 and **** *p* < 0.0001.

Propolis treatment was shown to increase the differentiation of MDSC [10] and we found a significant increase in the frequencies of mononuclear and polymorphonuclear cells in the BALF of animals treated with ArtC (Figure 2c). Next, we assessed the ability of ArtC to induce the differentiation of MDSC in vitro. MDSC is a heterogeneous population of immature myeloid cells that expands in pathologic situations and exhibits suppressive functions that regulate the immune response [19,20]. The two subsets of MDSC, granulocytic (PMN-MDSC: Ly6G^+^Ly6C^int^) and monocytic (M-MDSC: Ly6G^−^Ly6C^+^) were characterized as depicted in Figure 4a. ArtC was not toxic at concentrations up to 20 μM (Figure 4b), and therefore, for these experiments we used the same concentration of ArtC employed in Treg cell cultures (10 μM) (Figure 3b). The presence of ArtC increased the frequency of M-MDSC and reduced the frequency of PMN-MDSC compared to control cells (bone marrow cells cultured with IL-6 plus GM-GSF only) (Figure 4c–e).

**Figure 5:**
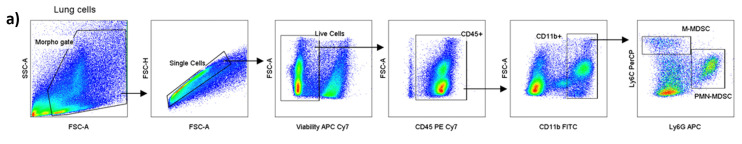

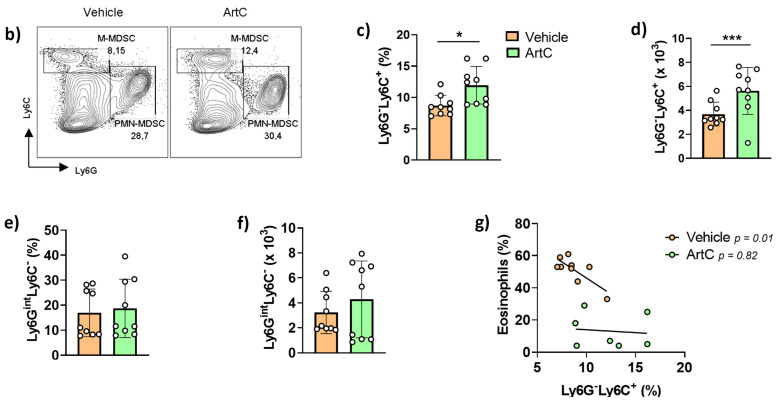
ArtC induces M-MDSC in the lungs of mice exposed to the allergen. (**a**) Gate strategy for Ly6G^−^Ly6C^+^ (M-MDSC) and Ly6G^+^Ly6C^int^ (PMN-MDSC) characterization in vivo; (**b**) representative dot plot of Ly6G^−^Ly6C^+^ and Ly6G^+^Ly6C^int^ in the lungs of mice exposed to the allergen treated with or not (Vehicle) with ArtC; (**c**) frequency, and (**d**) absolute number of Ly6G^−^Ly6C^+^ in the lungs; (**e**) frequency, and (**f**) absolute number of Ly6G^+^Ly6C^int^ in the lungs; (**g**) correlation between BALF eosinophils and lung Ly6G^−^Ly6C^+^. Data are representative of two independent experiments (n = 4–5/group/experiment) and expressed by mean ± SD. * *p* < 0.05 and *** *p* < 0.001.

We also evaluated the frequency of Ly6G^−^Ly6C^+^ (M-MDSC) and Ly6G^+^Ly6C^int^ (PMN-MDSC) in the lungs of mice exposed to the allergen, treated or not treated with ArtC. We used the gate strategy depicted in Figure 5a to characterize subsets of MDSC in the lungs. ArtC-treated mice exhibited an increase in the frequency (Figure 5b,c) and in the numbers (Figure 5d) of Ly6G^−^Ly6C^+^ cells but neither in the frequency (Figure 5b,e) nor in the number (Figure 5f) of Ly6G^+^Ly6C^int^ cells in the lungs. We performed a correlation analysis and found a negative correlation between the frequencies of pulmonary eosinophils and Ly6G^−^Ly6C^+^ cells in vehicle-treated mice, while no significant correlation was observed in the ArtC-treated group (Figure 5g). These findings suggest that Ly6G^−^Ly6C^+^ cells negatively regulate eosinophilic inflammation during allergic asthma.

### 3.5. M-MDSC Suppress the Allergic Airway Inflammation 

**Figure 6:**
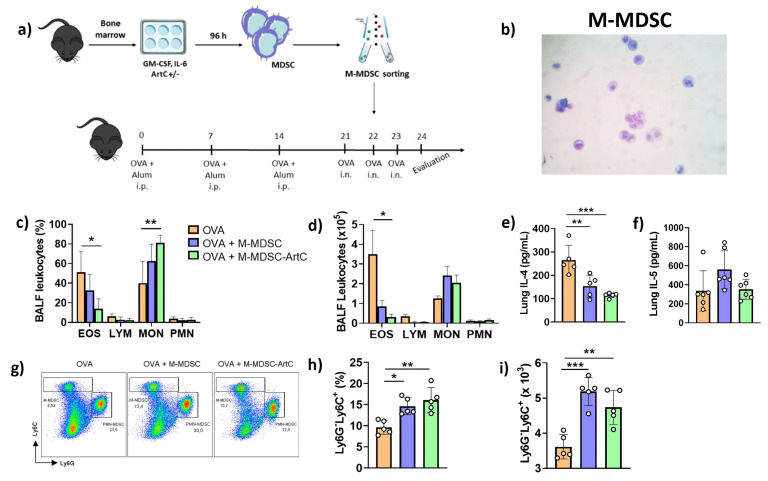

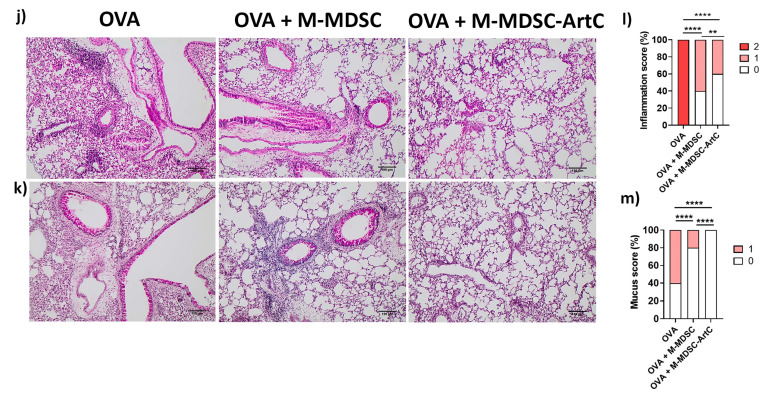
ArtC-induced M-MDSC reduces the allergic airway inflammation in mice. (**a**) Experimental design; (**b**) sorted M-MDSC, before cell transfer; (**c**) frequency and (**d**) number of eosinophils (EOS), lymphocytes (LYM), mononuclear cells (MON) and polymorphonuclear cells (PMN) in the BALF post cell transfer; (**e**) IL-4 and (**f**) IL-5 levels in the lungs; (**g**) representative dot plot of M- and PMN-MDSC in the lungs of post cell transfer; (**h**) percentage and (**i**) number of M-MDSC in the lungs; (**j**) representative images of lung inflammation (magnification 200×, scale bar 100 µM) and (**k**) mucus production (magnification 200×, scale bar 100 µM); (**l**) Lung inflammation score (0—without inflammation; 1—mild; 2—moderate to severe inflammation); (**m**) Mucus score (0—without mucus secretion; 1—mild). Data are representative of two independent experiments (n = 5–6/group/experiment), except for (**e**–**h**) (one representative experiment), and expressed by mean ± SD. * *p* < 0.05, ** *p* < 0.01, *** *p* < 0.001 and **** *p* < 0.0001.

To confirm the role of M-MDSC differentiated in the presence of ArtC in down-regulating allergic pulmonary inflammation, we next adoptively transferred sorted M-MDSC generated in vitro from bone marrow in the presence or absence of ArtC. On the second day of OVA challenge, sorted M-MDSC were transferred by intrapharyngeal route (Figure 6a). A representative photomicrography of sorted differentiated M-MDSC before cell transfer is shown in Figure 6b.

A significant reduction in both the frequency and number of eosinophils in the BALF of challenged mice was observed following ArtC-treated M-MDSC transfer (Figure 6c,d). Although a similar trend was observed following transfer of non-treated M-MDSC, the results were not significant (Figure 6c,d). An increase in the frequency, but not number, of monocytic cells was also observed following transfer of ArtC-treated M-MDSC (Figure 6c,d). In addition, we observed a reduction in IL-4 (Figure 6e), but not in IL-5 levels (Figure 6f) in the lungs homogenates after the transfer of ArtC-treated M-MDSC. IL-13 levels in the BALF and lungs homogenates are not changed between three groups (data not shown). Similarity, we did not observe any change in IL-10 levels in the lungs (data not shown). IL-4, IL-5 and IL-10 levels are not detected in the BALF. Flow cytometry analysis showed that intrapharyngeal transfer of sorted M-MDSC increased that cell population in the lung tissue compared with the lungs of mice that did not receive cell transfer (Figure 6g–i). Histological analysis revealed that transfer of both M-MDSC groups significantly decreased pulmonary infiltrates (Figure 6j) and mucus secretion (Figure 6k). However, the inflammation and mucus score (Figure 6l,m) were significantly lower in the lungs of mice transferred with ArtC-treated M-MDSC in comparison to those that received non-treated M-MDSC. These results confirm that ArtC treatment of M-MDSC increases their capacity in reducing eosinophil infiltration, inflammation, and mucus secretion in the lungs during airway allergic inflammation.

## 4. Discussion

In this study, we show for the first time that ArtC, a major component of green propolis [15], induced M-MDSC in vitro differentiation and augmented the frequency of lung M-MDSC (CD11b^+^Ly6G^−^Ly6C^+^) in an already established experimental model of asthma. The reduction in eosinophilic recruitment, pulmonary inflammation and mucus secretion, and the increase in M-MDSC frequency encourage the use of ArtC as a target molecule to be investigated as an adjuvant therapy for allergic asthma, which has not been previously pursued.

MDSC were primarily described in the context of tumors. Researchers observed myeloid hyperplasia in the tumor microenvironment, and later they identified that those cells displayed an immunosuppressive role [28]. Since then, the role of MDSC in tumors and other diseases has been explored. In the context of allergic asthma, MDSC can directly suppress the function of effector T (Teff) cells or induce Treg cells expansion in the lungs [29]. Cao and coworkers showed that PMN-MDSC reduced the allergic airway inflammation by inhibiting cytokine production and type 2 innate lymphoid cell (ILC2) function in a model of papain-induced lung inflammation [30]. Moreover, a previous study of our group showed that the treatment of animals exposed to allergen with green propolis induced accumulation of PMN-MDSC in lungs [10]. However, the role of M-MDSC in asthma and other allergic disorders was not known.

ArtC have been reported as an anti-inflammatory molecule [16,17,31]. Because our group previously showed that propolis treatment during experimental asthma induced Treg cells and MDSC, we considered it reasonable to investigate whether the mechanism of ArtC action would be the same as that of propolis. Although ArtC induced Treg cell differentiation in vitro, in vivo treatment with the compound failed to induce any increase in that population in the lungs of animals exposed to the allergen different from our group reported: an increase in Treg cells and PMN-MDSC in the lungs of mice exposed to the allergen and treated with propolis [10]. However, here we found an increase in M-MDSC in the lungs of mice treated with ArtC. These findings show that propolis and ArtC might play anti-inflammatory action through different mechanisms.

MDSC produce IL-10, TGF-β, and NO [22,32] and expresses specific markers related to their suppressive functions such as PD-L1 (Programmed Cell Death Ligand 1), *iNOS* (inducible Nitric Oxide Synthase), and Arg1 (Arginase 1) [23,33]. In vitro studies showed that blocking of Arg1 and iNOS increased CD8^+^ T and B cells proliferation [34,35], suggesting that these mediators were responsible for suppressor functions of MDSC. 

In allergic asthma, Cloots and coworkers showed that mice genetically deficient for Arg1 displayed a reduction in *Il4*, *Il5*, *Il13*, *Ccl2*, and *Ccl11*, all Th2-related genes [36], suggesting that Arg1 may regulate type 2 inflammation. Additionally, Arg1 expression is involved in airway fibrosis and bronchial hyper-reactivity [37]. Treatment with ArtC in vitro induced the expression of Arg1 in M-MDSC (data not shown). However, animals exposed to the allergen and treated with ArtC exhibited a reduction in Arg1 expression in lung M-MDSC (data not shown). Besides the reduction in Arg1 expression, ArtC-treated mice also displayed increased expression of PD-L1, although not significant, on M-MDSC in lungs (data not shown). PD-L1 is a co-inhibitory molecule expressed by several cell types. PD-L1 binds PD-1 receptor expressed in activated T lymphocytes and induces cell death and immune suppression [38]. Thus, the PD-L1-PD-1 interaction is also a possible mechanism by which ArtC-induced M-MDSC could negatively modulate Th2 cells in allergic asthma. 

Although M-MDSC transfer experiments confirmed the mechanism of action of ArtC inducing anti-inflammatory action in asthma, it remains to be investigated the exact mechanism by which M-MDSC reduces the eosinophilic influx and pulmonary inflammation in animals exposed to the allergen. These mechanisms might require Arg1, PD-L1, or other receptors or molecules that deserve further investigation. 

Previous studies reported that after the adoptive transfer of MDSC to mice exposed to the allergen, those MDSC migrate to the lungs and suppress inflammation by a mechanism dependent on TGF-β production [39,40]. Moreover, PMN-MDSC transfer was shown to inhibit lung inflammation, Th2-cytokine production, and ILC2 function by a mechanism dependent on COX1 expression [30]. Similarly, COX1 can be critical to PMN-MDSC expansion and function since COX1 knockout mice and aspirin-treated mice did not develop decreased lung inflammation after adoptive transfer [41]. However, to our knowledge, this is the first study that shows the anti-inflammatory potential of ArtC, a purified natural compound from propolis, in the context of allergic airway inflammation. Thus, ArtC can be selected as a target for adjuvant therapy for allergic asthma, based on its capacity to reduce eosinophilic influx, pulmonary inflammation, and mucus secretion via enhancement of M-MDSC anti-inflammatory functions.

## Figures and Tables

**Table 1:** Monoclonal antibodies used in flow cytometry assays.

Cell Type	Antibody	Fluorochrome	Clone
In vitro MDSC	CD11b	PE-Cy7	M1/70
	Ly6C	PerCP Cy5.5	AL-21
	Ly6G	APC	1A8
In vitro Treg cell	CD4	BB700	RM4-5
	Foxp3	PE	MF23
In vivo MDSC	CD45	PE-Cy7	30-F11
	CD11b	FITC	M1/70
	Ly6C	PerCP Cy5.5	AL-21
	Ly6G	APC	1A8
In vivo Treg cell	CD45	PE-Cy7	30-F11
	CD4	BB700	RM4-5
	Foxp3	PE	MF23
